# Metastatic seminoma and grade 1 follicular lymphoma presenting concurrently in a supraclavicular lymph node: a case report

**DOI:** 10.4076/1757-1626-2-7273

**Published:** 2009-08-19

**Authors:** Eric Jacobsen, Jey-Hsin Chen, Brian Schurko, Carol Benson, William K Oh

**Affiliations:** 1 Mount Sinai School of MedicineOne Gustave L. Levy Place - Box 1079 New YorkNY 10029, USA; 2 Massachusetts General HospitalBoston, MA, 55 Fruit St, Boston, MA 02114USA; 3 Brigham and Women’s HospitalBoston, MA, 75 Francis St, Boston, MA 02115USA

## Abstract

An asymptomatic 67-year-old man presented with a left supraclavicular lymph node that enlarged over a 2-month period which was biopsied. Pathologic features were consistent with involvement by metastatic seminoma and follicular lymphoma, follicular pattern, grade 1 (of 3). Staging Positron Emission Tomography/Computed Tomography scans indicated several areas of enlarged lymph nodes. The patient completed chemotherapy with bleomycin, etoposide, and cisplatin chemotherapy. This is the first reported case of metastatic seminoma and follicular lymphoma occurring in the same lymph node. No obvious pathophysiologic link exists between these two malignancies and there are no shared common risk factors. Given the natural history of these two malignancies, if this patient develops recurrent lymphadenopathy, it will be difficult to identify whether the enlarged lymph nodes represent recurrent seminoma or follicular lymphoma without a biopsy of each pathologically enlarged node. Similarly, Fluorodeoxyglucose- Positron Emission Tomography is known to be active in both seminoma and follicular lymphoma, making this scan non-specific in this patient. Finally, this patient had no baseline elevation in any germ cell tumor marker. Thus, serum tumor markers cannot be relied upon as surrogates for response to chemotherapy or as identifiers of relapsed seminoma.

## Case presentation

An asymptomatic 67-year-old white, American man presented with a left supraclavicular lymph node that enlarged over a 2-month period. His primary care physician recommended a biopsy.

**Figure 1.**
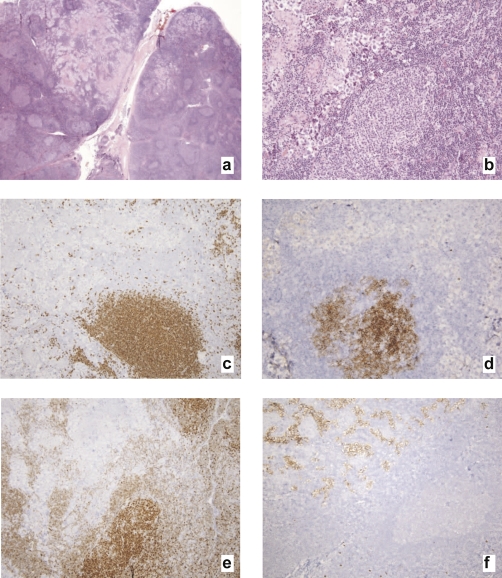
Lymph node, metastatic seminoma and follicular lymphoma. **(a)** Hematoxylin and eosin, low-power magnification. **(b)** Hematoxylin and eosin, high-power magnification. **(c)** Immunoperoxidase stain for CD20. **(d)** Immunoperoxidase stain for CD10. **(e)** Immunoperoxidase stain for bcl-20. **(f)** Immunoperoxidase stain for c-kit.

The left supraclavicular lymph node was partially effaced by neoplastic nodules of small lymphocytes with cleaved nuclei and scant cytoplasm intermixed with rare centroblasts, and by metastatic seminoma which is associated with a non-necrotizing granulomatous reaction (Figure 1a, b). The neoplastic lymphocytes were positive for CD20 (Figure 1c) and CD10 (Figure 1d) and showed monotypic surface immunoglobulin lambda light chain expression by flow cytometric analysis. The anti-apoptotic marker bcl-2 (Figure 1e) was reactive in a subset of the follicles. The seminoma cells were positive for c-kit (Figure 1f) and negative for keratin and for melanocytic and lymphocytic markers (not shown). The overall features were consistent with involvement by metastatic seminoma and follicular lymphoma, follicular pattern, grade 1 (of 3).

The patient had no fevers, chills, night sweats, unintentional weight loss, or other systemic symptoms. He had not noted any testicular masses or pain. He was not experiencing shortness of breath or cough. His past medical history was notable only for benign prostatic hypertrophy, with a prostate biopsy showing no evidence of malignancy, and Dupuytren’s contracture of the right hand.

Physical examination was notable for a healed left supraclavicular lymphadenectomy scar, no other palpable lymph nodes and a slightly atrophic left testicle with no palpable testicular masses. A serum prostate-specific antigen (PSA) was 3.23 ng/ml, serum beta-human chorionic gonadotrophin (HCG) was undetectable, and a serum alpha-fetoprotein (AFP) was 4.2 ng/ml (normal range 0-7.6 ng/ml). Serum LDH was 389 u/l (normal range 313-618 u/l). A complete blood count with differential, serum electrolytes, renal function, and liver function were all within normal limits.

A scrotal ultrasound demonstrated a mildly heterogeneous and small (2.6 × 1.3 × 2.2 cm) left testicle consistent with atrophy as well as a small left varicocele. The right testicle was normal (3.6 × 2.5 × 1.8 cm). Positron emission tomography (PET) and computed tomography (CT) scan performed approximately one month after the original CT scan demonstrated FDG uptake in left supraclavicular (1.5 × 0.8 cm), bilateral axillary (1.6 × 1.0 cm on left, 1.5 × 1.1 cm on right), anterior mediastinal (without CT correlate), retroperitoneal (1.5 × 1.9 cm), and mesenteric (1.6 × 1.0 cm) lymph nodes. The prostate gland was slightly enlarged and exhibited mild FDG uptake.

This is the first reported case of metastatic seminoma and follicular lymphoma occurring in the same lymph node. We are aware of a report of metachronous seminoma and Hodgkin’s disease [1]. No obvious pathophysiologic link exists between these two malignancies and there are no shared common risk factors. The patient completed chemotherapy with bleomycin, etoposide, and cisplatin (BEP) and remains in complete remission over three years later [2]. He had no specific testicular primary noted, but did have an atrophic left testicle which could have represented a “burnt out” primary. Although his lymphoma did not require therapy, these agents are also active in lymphoid malignancies [3]. This may make evaluation of response and monitoring for relapse difficult, as both seminoma and follicular lymphoma would be expected to respond to BEP. However, follicular lymphoma will inevitably relapse after chemotherapy whereas seminoma has a high cure rate in this setting. If this patient develops recurrent lymphadenopathy, it will be difficult to know whether the enlarged lymph nodes represent recurrent seminoma or follicular lymphoma without a biopsy of each pathologically enlarged node. Similarly, FDG-PET is known to be active in both seminoma and follicular lymphoma, making this scan non-specific in this patient [4,5]. Finally, this patient had no baseline elevation in any germ cell tumor marker. Thus, serum tumor markers cannot be relied upon as surrogates for response to chemotherapy or as identifiers of relapsed seminoma.

## Abbreviations

FDG: fluorodeoxyglucose
HCG: Human chorionic gonadotropin
LDH: lactate dehydrogenase
PSA: prostate specific antigen
